# Optimizing livestock carrying capacity for wild ungulate-livestock coexistence in a Qinghai-Tibet Plateau grassland

**DOI:** 10.1038/s41598-021-83207-y

**Published:** 2021-02-11

**Authors:** Yueheng Ren, Yanpeng Zhu, Davide Baldan, Mengdi Fu, Bin Wang, Junsheng Li, Anping Chen

**Affiliations:** 1 State Environmental Protection Key Laboratory of Regional Eco-process and Function Assessment, Chinese Research Academy of Environmental Sciences, Beijing, 100012 China; 2 Department of Biology, University of Nevada, Reno, NV 89557 USA; 3 College of Life Sciences, Hunan Normal University, Changsha, 410006 China; 4 Department of Biology and Graduate Degree Program in Ecology, Colorado State University, Fort Collins, CO 80523 USA

**Keywords:** Agroecology, Conservation biology, Ecosystem services, Grassland ecology

## Abstract

Wild ungulates are an important part of terrestrial ecosystems and play a critical role in maintaining ecosystem health and integrity. In many grassland ecosystems that are habituated by wild ungulates, the coexistence of domestic ungulates has created a conflict over grazing resources. Solving this conflict requires a balanced and sustainable policy that satisfies both the needs of wildlife protection and food production. Here, we assess the optimal grassland livestock carrying capacity of an alpine grassland on the Qinghai-Tibet Plateau, given the coexistence of wild populations of kiangs (*Equus kiang*) and Tibetan gazelles (*Procapra picticaudata*), two key species grazing in this region. We use kriging and the MaxEnt method to estimate the population sizes of kiangs and Tibetan gazelles in Maduo County, Qinghai Province. We then convert the estimated population size of the two species into sheep units and calculate the residual carrying capacity for livestock grazing. We show that after accounting for the grazing need for kiangs and Tibetan gazelles, grassland in Maduo is capable of supporting 420,641 sheep units, which is slightly more than the current livestock population. However, the residual carrying capacity is highly uneven across the region, and overgrazing is found in many areas of Maduo, especially in northern Maduo. This research provides a useful framework for planning sustainable livestock farming for the Qinghai-Tibet Plateau and other regions facing wildlife-livestock conflict.

## Introduction

Grassland ecosystems cover approximately 26% of the global land area and sustain most of the world’s livestock^1,2^. These ecosystems also accommodate many wildlife species, including grazing herbivores. Competition between livestock and grazing wildlife for food, water, and space is therefore a common issue in grassland ecosystem and biodiversity conservation practices worldwide^3–5^. For example, competition between livestock and the kiang (*Equus kiang*) threatens the conservation prospects of the kiang in the Ladakh of the Trans-Himalaya region^6^, while conflict over sheep ranching is considered a primary cause of the population decline of guanacos (*Lama guanicoe*) in southern Chile^7^. Due to the increasing demand for beef and lamb, livestock populations have steadily grown over the past decades, resulting in overgrazing in many grasslands^8^. Livestock overpopulation and overgrazing have significantly lessened resource availability for wildlife and, in some cases, have degraded grassland quality to the point that desertification has occurred in some arid and semiarid grasslands^9,10^. Degraded grasslands usually have reduced forage production for both livestock and wildlife, suffer from severe water and soil loss and are more vulnerable to environmental changes^11^. These changes can further intensify the conflict between livestock and wildlife and are largely responsible for the decreasing wildlife population and the endangerment of many species^12^.

For sustainable animal husbandry and long-term coexistence between wildlife and livestock to occur, livestock overpopulation must be minimized; thus, a reasonable carrying capacity for livestock must be quantified^13^. Quantifying livestock carrying capacity is a key issue, with a wide number of techniques and studies available^14^. However, most studies focus only on the bottom-up limitations of grassland productivity and water availability on livestock carrying capacity, such as grass yield, livestock feeding intake, grazing utilization rate, key area selection, climate influence, and supplementary feeding^15–18^. Few of them consider wildlife conservation in the quantification of livestock carrying capacity, even in areas with strong wildlife-livestock conflict. However, excluding livestock entirely to protect indigenous wildlife may be difficult to implement in areas where indigenous people raise animals for sustenance^19^. A balance between wildlife conservation and existing animal husbandry is, however, possible with careful planning that considers the needs of both.

The Three-River-Source area of the Tibetan Plateau in China is a region where wild ungulate species and ungulate-based husbandry coexist. Located at the headwaters of three major river systems—the Yangtze, Yellow and Lancang rivers—and lying, for the most part, 4000 m above sea level, the Three-River-Source area is dominated by alpine steppes and meadows^20^. This region performs vital functions, such as food and fiber supply, soil and water conservation, and carbon sequestration, and is an important habitat for a variety of endangered wildlife species^21^. The region is also listed as one of the “Global 200” priority ecoregions of global significance in conservation^22^ and is a hotspot for global warming (second only to the Arctic region)^23^. Nomadic herding and animal husbandry are the main industries in the region. In the past, the traditional grazing methods of local herders coexisted with herbivorous wildlife. However, since the 1960s, animal husbandry has increased in this area, resulting in serious overpopulation and overgrazing. This uncontrolled increase in livestock has negatively affected both the welfare of wildlife and grassland ecosystems^24,25^. In 2003, the Three-River-Source area was designated as a National Nature Reserve (then later planned as a National Park), and strict protection and restoration measures were planned and implemented. Among these protection measures, the reduction or even the complete ban of livestock grazing is a top priority. However, preserving the cultural heritage and traditional lifestyle of indigenous herders and allowing for sustainable development of the area are also both important goals for the Three-River-Source National Park Master Plan^26^. Achieving a balanced resolution between these conflicting goals requires an improved quantification of the wildlife populations and their foraging demands. With this information, we can then estimate the carrying capacity for livestock.

**Figure 1:**
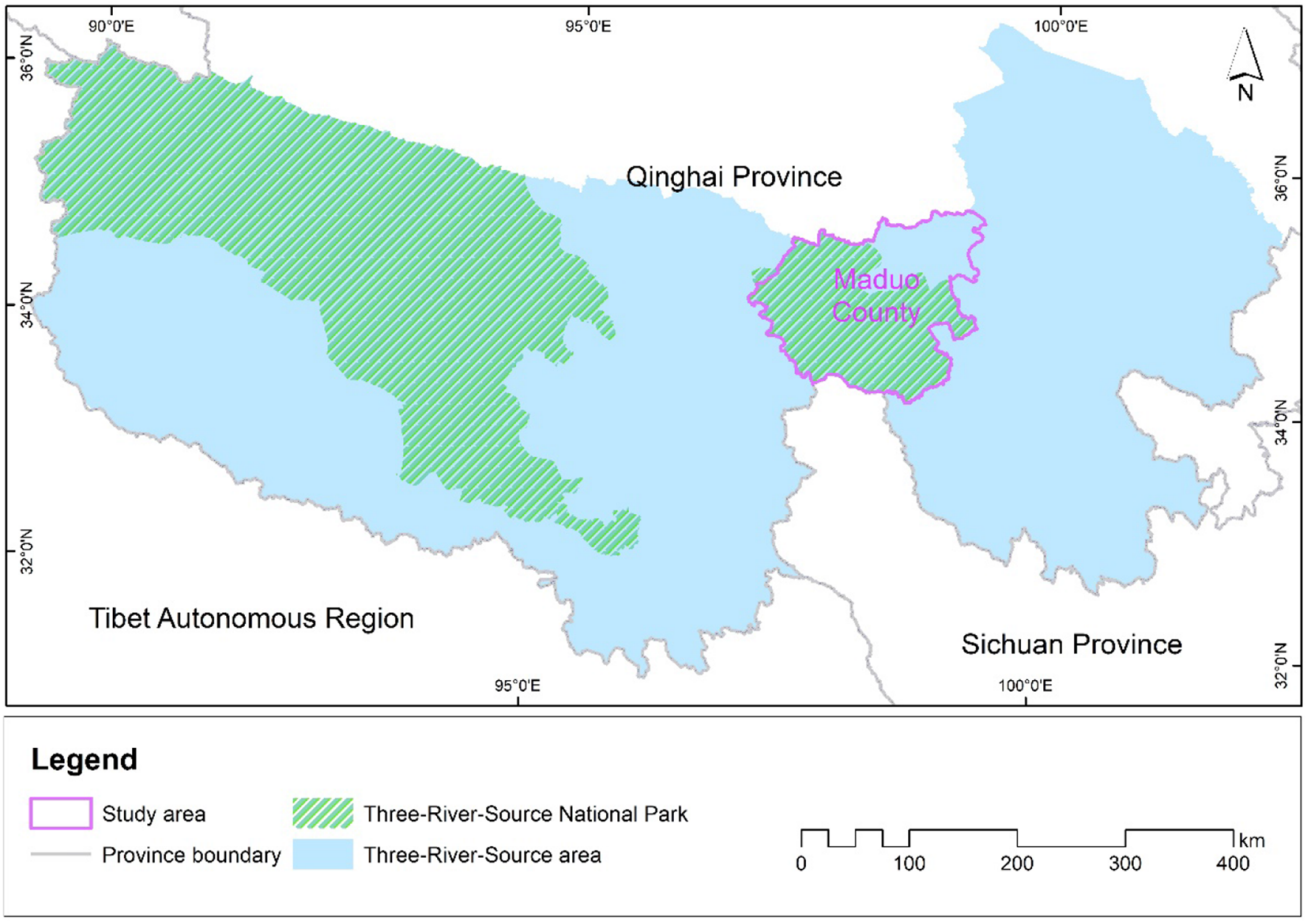
The spatial location of the study area, the Three-River-Source National Park and the Three-River-Source area. The map was generated using ArcGIS 10.2, https://desktop.arcgis.com/en/.

Here, we choose Maduo County, one of the 17 counties in the Three-River-Source area, as the target region to conduct population research on wild ungulates to determine the region’s livestock carrying capacity (Fig. 1). This is to ensure a sustainable coexistence of both wild ungulates and livestock. Our goal is to then develop an applicable strategy that simultaneously considers both the needs of local herders and the protection of wildlife populations and grassland ecosystems. While the study uses Maduo County as a specific example, the framework we develop here will provide important insights for the entire Qinghai-Tibet Plateau and other regions facing similar competing demands for wildlife protection and livestock production.

## Results

**Figure 2:**
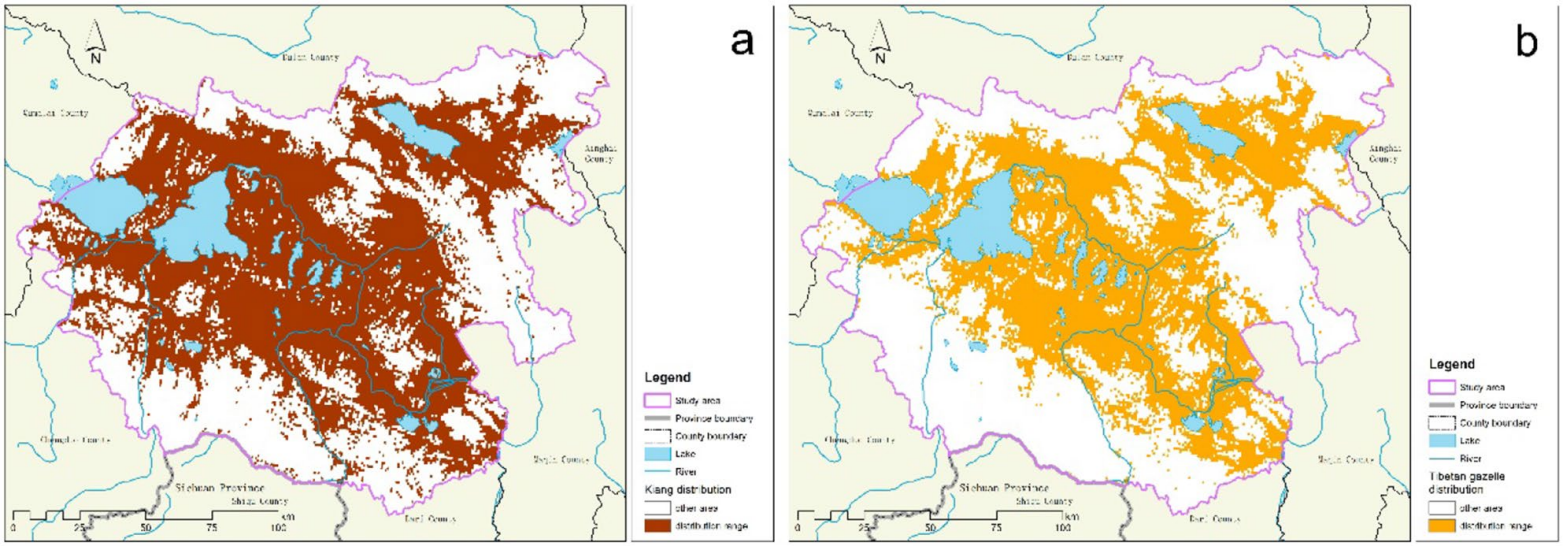
The distribution ranges of kiangs (**a**) and Tibetan gazelles (**b**) in Maduo County. The map was generated using ArcGIS 10.2, https://desktop.arcgis.com/en/.

**Figure 3:**
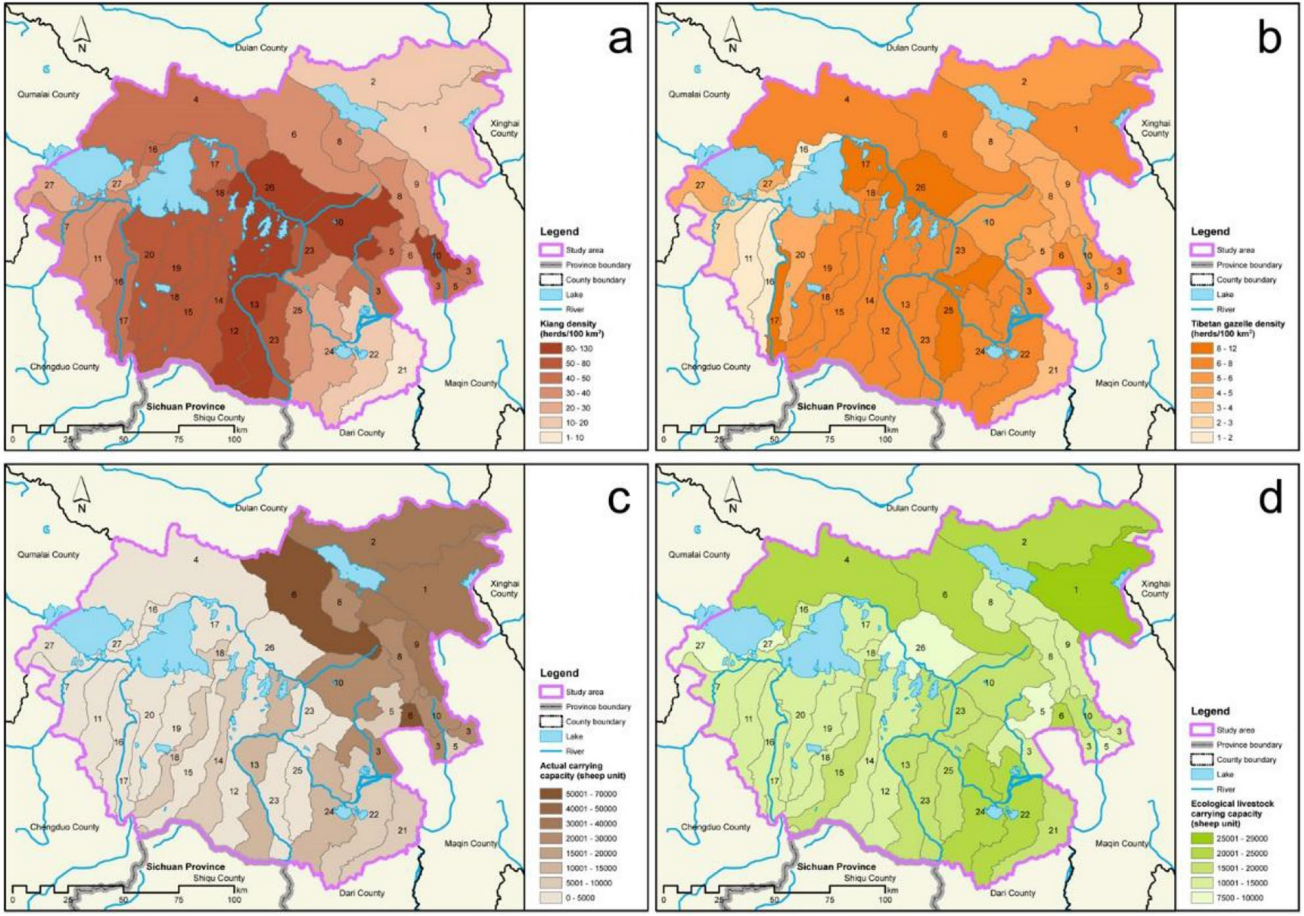
The density spatial distribution of kiangs (**a**) and Tibetan gazelles (**b**), the actual carrying capacity (**c**) and the ecological carrying capacity (**d**) of Maduo County. Maps were produced in ArcGIS 10.2 (https://desktop.arcgis.com/en/) based on shapefiles and data collated for this study, and Adobe Illustrator was used to combine them into one figure.

Based on field surveys and Maxent model simulations, we estimate that the suitable area for kiangs (*Equus kiang*) is 12,650 km^2^ in Maduo County, and that of Tibetan gazelles (*Procapra picticaudata*) is 9897 km^2^. Overall, we estimate the presence of 11,397 kiangs and 1545 Tibetan gazelles in Maduo, equal to 68,834 sheep units. Both species are widely distributed across the county (Fig. 2). The population density of kiangs is high in northern Zhaling Lake and Eling Lake and in the central and south-central parts of Maduo County (Fig. 3a). The population density of Tibetan gazelles is also high in northern Zhaling Lake and Eling Lake (Fig. 3b), making this region a hotspot for both wild ass and gazelle distributions.

A household livestock survey provided by the Three-River-Source National Park Administration indicates that there were 338,159 sheep units (73% cattle, 24% sheep, 3% horses) in Maduo County in 2015. The distribution of livestock density is uneven, with the highest population density found south of Lake Donggeicuona (land parcel #6), followed by northeastern Maduo (Fig. 3c). The satellite-derived total annual grass yield in Maduo County is estimated to be approximately 1,293,500 tons. This grass yield quantity is estimated to support 489,474 sheep units (theoretical total carrying capacity). Factoring in wildlife, the ecological livestock carrying capacity of Maduo County’s grassland would be 420,641 sheep units. The spatial distribution of ecological livestock carrying capacity (Fig. 3d) shows a high carrying capacity in the northeastern part of the region but a low carrying capacity in the southwestern part of the region. In particular, the lowest ecological livestock carrying capacity is found at land parcel #26 (Fig. 3d), where a high population density of kiangs is observed (Fig. 3a).

**Figure 4:**
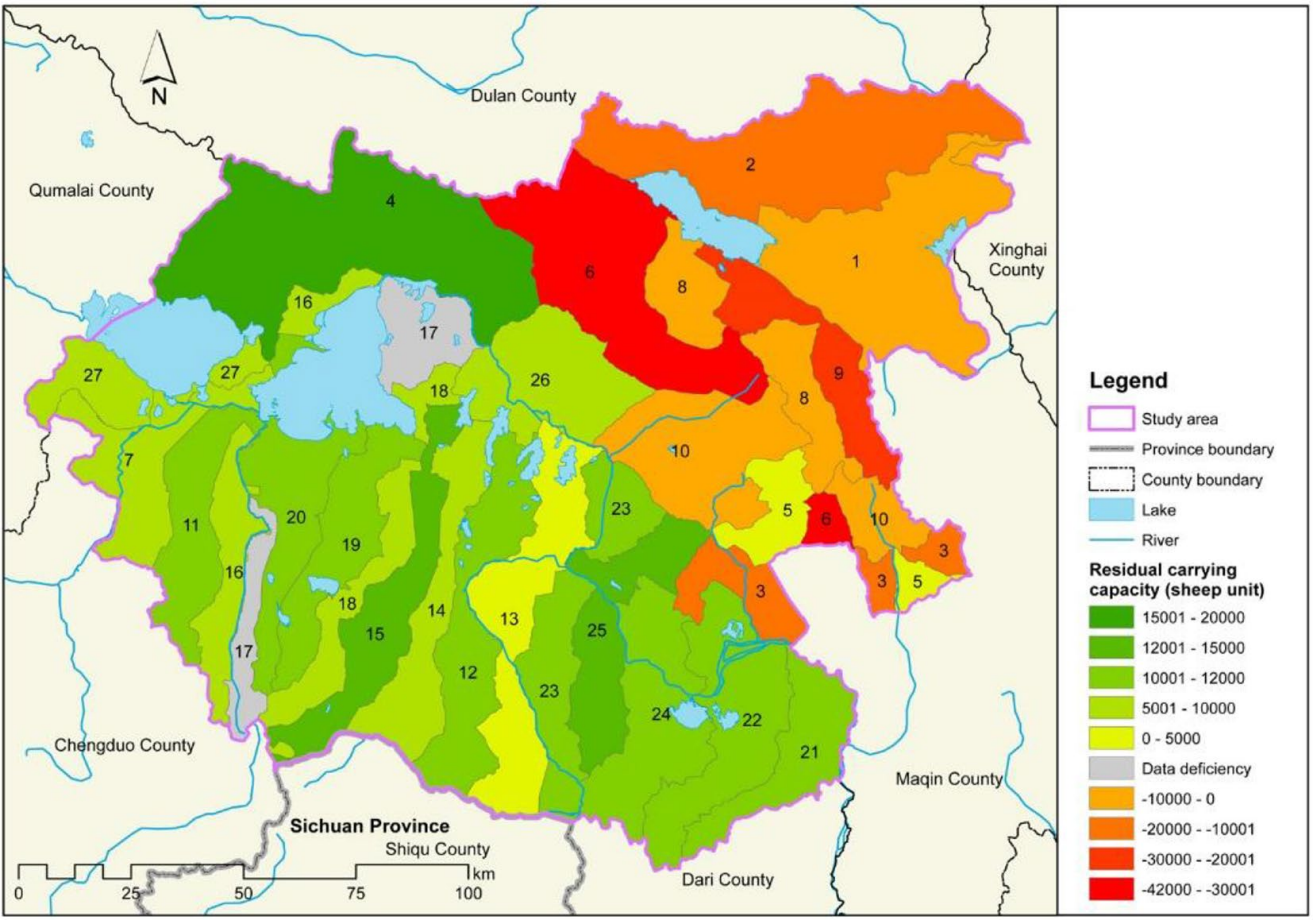
Residual carrying capacity in Maduo (with two wild ungulate species). The map was generated using ArcGIS 10.2 (https://desktop.arcgis.com/en/) based on shapefiles and data collated for this study.

By subtracting the actual carrying capacity of each land parcel from the ecological livestock carrying capacity, we obtain the residual carrying capacity (RCC) for each parcel, which is a total of 82,482 sheep units (Fig. 4). The RCC is positive in the southwestern part of Maduo County, indicating that the area is capable of feeding more livestock without threatening the existing ungulate wildlife. In contrast, northeastern Maduo County has negative RCC values and an overloaded livestock population. Among the land parcels, land parcel #6 is most overloaded with livestock. Supplementary Table S1 provides summary statistics on wild ungulates and livestock carrying capacity for each land parcel.

## Discussion

The Three-River-Source National Park (TNP) is China’s first national park and represents an experiment to induce a major shift in Chinese conservation efforts. When planning conservation efforts in the TNP to protect the broad alpine grassland socioecosystem, both the needs of wildlife habitats and the nomadic lifestyle of the indigenous people must be considered^27^. Here, we present an analysis of how wildlife and livestock can share grass yields in Maduo, a key region within the TNP. Our analysis shows that the current livestock population in Maduo is below the carrying capacity of the region after considering the foraging needs of the two main native herbivores: the kiang and the Tibetan gazelle. However, the spatial distribution of the residual carrying capacity is highly uneven, and some areas have negative RCC values (indicating overloading). Thus, our results indicate that, at least for Maduo, it is possible to reach sustainable coexistence between livestock and key wildlife species given appropriate planning.

The mapping of the grassland carrying capacity also provides a useful tool for guiding husbandry planning within the TNP (Fig. 4). Specifically, for overloaded land parcels, the priority should be to sustain or even increase the wildlife population in accordance with the TNP master plan^26^. Furthermore, the livestock density should be kept below the ecological carrying capacity. Different livestock reduction goals, timelines, and measures should be established in accordance with the requirements of the functional zoning of the national park. For other overloaded sites outside of the national park, the priority should be to ensure the sustainability of grassland productivity. Accordingly, different management tools need to be adopted to ensure that the livestock density is below the ecological carrying capacity. Specific measures that should be taken in land parcels #1, #2, #3, #6, #8, #9 and #10 include (1) livestock reduction and ecological compensation. Setting a limit on livestock populations based on the ecological carrying capacity and reducing livestock populations accordingly. Afterwards, ecological compensation is used. These measures are suitable for land parcels #6 and #9, which have relatively serious overgrazing problems. (2) Developing alternative industries for grazing. Ecological tourism, traditional handicraft and the ethnic culture industry could be developed to maintain residents’ income, especially in land parcels #3, #6, #8 and #10, which are partially located in the TNP. (3) Optimizing grazing patterns. Establishing an ecological animal husbandry cooperative could break the original boundary of land parcels. Then, implementing rotational grazing and seasonal rest grazing in a larger area can restore grassland productivity. In addition, the removal of boundary fences and avoidance of areas where ungulates gather for rotational grazing can help protect wild ungulates^28^.

The protection of wildlife and ecological integrity often requires native residents to relocate or to change their field of work. It is therefore useful to provide financial compensation to incentivize cooperation from the local people^29^. However, determining appropriate compensation standards in the pursuit of protecting wildlife has always been challenging^30,31^. Our work on the ecological carrying capacity provides a useful framework in this direction. In particular, the GIS-based carrying capacity estimation makes the compensation implementable at the land parcel level. This method is also applicable to other grassland and meadow ecosystems where livestock and wild ungulates coexist, as in many other regions of the Qinghai-Tibet Plateau^32^, East Africa^33^, and Patagonia in South America^7^. With the aim of protecting wild ungulates, our study provides a reference for reducing the costs of protecting the environment, achieving a balance between wildlife and livestock, and formulating programs to financially compensate locals for livestock reduction. Furthermore, our framework can also be extended to other ecosystem types that face similar resource competition between the livelihood of locals and wildlife protection, such as forest^34^, coastal^35^ and oceanic^36^ ecosystems.

It is important to note that our method might be subject to some limitations. First, estimating carrying capacity based on the current grazing utilization rate (65%)^37^ may overestimate the actual capacity of livestock and wild ungulates. Previous studies have suggested that the current grassland grazing rate in many areas of the Qinghai-Tibet Plateau, including Maduo, is not sustainable and has caused grassland degradation^25,38^. To reverse this grassland degradation, it would be necessary to further reduce the number of livestock. Second, we considered only kiangs and Tibetan gazelles, the two dominant wild ungulate species in this study. Although rare and not major competitors with livestock, other grazing species, such as Tibetan antelope and wild yak, are found in the region. Future research with more extensive field surveys may include the protection of those rare grazing species as well. Third, our NDVI-based forage production estimation neglects the potential difference in edible forages of different plant communities or grasslands with different health conditions. This fixed average forage ratio has been widely used in previous studies^39,40^ but could still be problematic, especially when the plant community shifts under climate change, e.g., from C_4_-dominant grasslands to C_3_-dominant grasslands with extreme drought^41^. Extensive field surveys of grazing preferences and edible proportions of different grassland plant communities, together with high-resolution remote sensing that can distinguish these different plant communities, are needed to improve our forage production estimation. Fourth, while the MaxEnt model driven by projected climate scenarios can also be used to predict future wild ungulate distributions^42^, current wildlife surveys are still insufficient to build such dynamic population models that are needed for a better understanding of long-term population dynamics. In particular, it is important to note that the distribution of wild ungulates in Maduo is also determined by anthropogenic factors that are difficult to factor into the model. Long-term repeated and standardized wildlife population surveys and monitoring are thus needed to provide standard basic data to include population growth goals in the estimation of ecological carrying capacity and to support wildlife conservation planning.

## Methods

### Study area

Maduo County is located in the eastern part of the Three-River-Source area (Fig. 1), at approximately 34° 0′ ~ 35° 40′ N and 96° 50′ ~ 99° 20′ E, with an area of ~ 25,300 km^2^. The topography of the county is relatively flat, and the elevation lies mostly between 4200 and 4800 m above sea level. The region has a typical highland continental climate with strong solar radiation and large daily temperature differences. The average annual temperature is − 3.8 °C, and the average annual precipitation is approximately 304 mm^43^. Vegetation types in Maduo include alpine steppe ecosystems, alpine meadow ecosystems, wetland ecosystems, and alpine desert ecosystems. The county lies in the source area of the Yellow River and has important water conservation functions. The county is divided into 27 land parcels according to the boundaries of traditional grazing management.

### Data sources

Two national protected species of China, both wild ungulates that prevail in the grasslands of Maduo, are considered in the study: the kiang, a protected species also listed in the Convention on International Trade in Endangered Species of Wild Fauna and Flora (CITES), and the Tibetan gazelle. The distribution and abundance data of these two species were obtained from surveys organized by the Three-River-Source National Park Administration in 2015 and 2016. These surveys recorded wild animals observed while traversing sample lines, including their species names, abundances, traces, and geographic location information. In total, they recorded 2900 kiangs with an average discovery rate of 1.33 kiangs per km per season and 1067 Tibetan gazelles with an average discovery rate of 0.49 Tibetan gazelles per km per season.

The climate data required to simulate the distribution of kiangs and Tibetan gazelles were downloaded from Worldclim (http://worldclim.org), and the topography data were obtained from the Geospatial Data Cloud (http://www.gscloud.cn). The spatial resolution of all the data is uniform at 30 arcseconds (approximately 1 km).

The livestock data of Maduo County, which were acquired through a household survey, were also provided by the Three-River-Source National Park Administration. All environmental and livestock data were for 2015.

### Species data processing

Ordinary kriging is used to extrapolate the distribution and abundance data of wild ungulates obtained from field surveys to the regional scale at a resolution of 5 × 5 km. Kriging interpolation is applicable to grassland and forest ecosystems with relatively continuous habitats^44,45^. In this study, based on the characteristics of the survey route settings, the species abundance density (SAD) was estimated for each survey line. The estimated SAD was then translated to the species abundance of each season at each 5 × 5 km grid using ArcGIS 10.2 and ordinary kriging^44^.

The species distribution model Maxent^46^ was used to simulate the potential distribution range of kiangs and Tibetan gazelles based on (1) the distribution of points of these two species and (2) environmental information such as elevation, slope, slope aspect, and climate variables. The potential distribution range is combined with the previously interpolated density distribution map and matched with the administrative land boundary of Maduo County to obtain simulated abundances of the two species distributed in each land parcel.

### Grass production data processing

Remote sensing has been widely used to estimate ecosystem production^47–49^. Here, we used MODIS MOD13Q1 data (https://lpdaac.usgs.gov/products/mod13q1v006/) to estimate the NDVI (normalized difference vegetation index) values of Maduo County from July to August 2015 (Supplementary Fig. S1). For each grid, we use the maximal value composite method to represent its NDVI and then use the equation derived from Lü et al. to calculate grass yield from NDVI^40^:

1 $$Y = - 47.021 + 440.21X,$$

where *Y* is the amount of grass production and *X* is the NDVI value.

### Theoretical livestock carrying capacity calculation

According to the standard *Calculation of rangeland carrying capacity* (NY/T635-2015) published by the Ministry of Agriculture and Rural Affairs of the People's Republic of China^50^, theoretical carrying capacity refers to the maximum number of livestock that can be supported by an area of grassland over a certain period while sustaining grass production. Based on Xin et al., we adopt the following formula to calculate the theoretical carrying capacity without considering grass use by wild animals for grassland use^37^:

2 $${C_{tp}} = \, \left( {Y \times E \times U} \right)/\left( {I \times T} \right)$$

where *C*_*tp*_ is the theoretical carrying capacity per unit area, *Y* is the grass yield per unit area, *E* is the edible forage ratio, *U* is the grazing utilization rate, *I* is the daily intake of livestock, and *T* is the number of grazing days. According to Zhao et al*.*, the edible forage ratio in Maduo County is 85%^51^. Xin et al. estimated that for alpine steppes and alpine meadows, the two dominant vegetation types in Maduo, the grazing utilization rate is 65% ~ 70%^37^. Here we assume it to be 65%. The daily intake per sheep unit is 4.0 kg, and the number of grazing days is 365 days^37^.

The grass consumption by wild ungulates can be calculated as:

3 $${C}_{w}=\sum_{i=1}^{n}\left({D}_{i}\times {S}_{i} \times {K}_{i }\right)$$

where *C*_*w*_ is the total number of wild ungulates in sheep units in each land parcel and *D*_*i*_, *S*_*i*_ and *K*_*i*_ represent the density, distribution area of each land parcel, and the rate that converts species *i* to standard sheep units. According to Lu et al., a kiang equals 6 sheep units, and a Tibetan gazelle equals 0.3 sheep units based on the weight ratio between its average weight (approximately 14 kg) and that of a standard sheep unit^32,52^.

After considering the grass consumption by wild ungulates, the carrying capacity (*C*_*t*_) of each land parcel is estimated as:

4 $${C_t} = {C_{tp}} \times S - {C_w}$$

where *C*_*t*_ is the ecological carrying capacity of a land parcel, and S is the land parcel area.

### Bioethics statement

The study does not involve any animal experiments or operations.

## Supplementary Information

Supplementary Information

## Data Availability

The MODIS MOD13Q1 data is available at https://lpdaac.usgs.gov/products/mod13q1v006/. Other data/code that support the findings of this study can be obtained from the authors upon reasonable request.
