# Supplementary material for: Optimizing livestock carrying capacity for wild ungulate-livestock coexistence in a Qinghai-Tibet Plateau grassland
Source: Sci Rep. 2021 Feb 11;11:3635. doi: 10.1038/s41598-021-83207-y (PMC7878488; doi:10.1038/s41598-021-83207-y)
Supplement: Supplementary file 1 — Supplementary Information [file 41598_2021_83207_MOESM1_ESM.docx]

**Optimizing livestock carrying capacity for wild ungulate-livestock coexistence in a Qinghai-Tibet Plateau grassland**

Yueheng Ren^1^, Yanpeng Zhu^1*^, Davide Baldan^2^, Mengdi Fu^1^, Bin Wang^3^, Junsheng Li^1*^, Anping Chen^4*^

^1^ Research Center for Biodiversity, Chinese Research Academy of Environmental Sciences, Beijing 100012, China.

^2^ Department of Biology, University of Nevada, Reno, NV 89557, USA.

^3^ College of Life Sciences, Hunan Normal University, Changsha 410006, China.

^4^ Department of Biology and Graduate Degree Program in Ecology, Colorado State University, Fort Collins, CO 80523, USA.

Correspondence should be sent to: Yanpeng Zhu (zhuyp@craes.org.cn), Junsheng Li (lijsh@craes.org.cn), or Anping Chen (anping.chen@colostate.edu)

**Supplementary Table S1** Livestock carrying capacity for each land parcel in Maduo County

| Land parcel number | Kiang density (herds / 100 km^2^) | Tibetan gazelle density (herds / 100 km^2^) | Wild ungulates sheep unit | Ecological carrying capacity (sheep unit) | Horse number | Cattle number | Sheep number | Actual carrying capacity (sheep unit) | Residual carrying capacity (sheep unit) |
| --- | --- | --- | --- | --- | --- | --- | --- | --- | --- |
| 1 | 11.11 | 6.36 | 1049 | 28923 | 108 | 7526 | 5452 | 36204 | -7279 |
| 2 | 19.72 | 5.42 | 2263 | 21793 | 213 | 6845 | 4998 | 33656 | -11864 |
| 3 | 48.92 | 7.93 | 1518 | 11654 | 195 | 5530 | 5980 | 29270 | -17618 |
| 4 | 49.10 | 7.79 | 6404 | 23042 | 12 | 521 | 2436 | 4592 | 18453 |
| 5 | 44.83 | 4.70 | 1055 | 7677 | 92 | 1102 | 2600 | 7560 | 116 |
| 6 | 35.18 | 6.77 | 3241 | 21438 | 182 | 10638 | 19638 | 63282 | -41843 |
| 7 | 31.52 | 2.58 | 1331 | 12632 | 15 | 528 | 1318 | 3520 | 9111 |
| 8 | 34.90 | 4.67 | 1631 | 13940 | 76 | 4719 | 1910 | 21242 | -7302 |
| 9 | 26.31 | 4.40 | 950 | 12638 | 111 | 6646 | 5825 | 33075 | -20439 |
| 10 | 92.57 | 5.86 | 6561 | 17345 | 76 | 4719 | 1910 | 21242 | -3896 |
| 11 | 34.63 | 1.49 | 1365 | 13868 | 16 | 520 | 658 | 2834 | 11033 |
| 12 | 120.71 | 8.25 | 6388 | 13910 | 29 | 294 | 1604 | 2954 | 10954 |
| 13 | 116.53 | 8.09 | 6948 | 16392 | 148 | 2262 | 3143 | 13079 | 3311 |
| 14 | 79.53 | 7.75 | 3196 | 13245 | 30 | 425 | 5576 | 7456 | 5789 |
| 15 | 52.47 | 6.98 | 2380 | 17242 | 32 | 278 | 1273 | 2577 | 14663 |
| 16 | 45.87 | 1.70 | 1569 | 12895 | 24 | 685 | 1207 | 4091 | 8806 |
| 17 | 42.71 | 10.32 | 1688 | 11809 | - | - | - | - | - |
| 18 | 59.11 | 8.16 | 2048 | 13314 | 24 | 400 | 4337 | 6081 | 7231 |
| 19 | 52.33 | 7.33 | 2024 | 14995 | 26 | 355 | 1951 | 3527 | 11466 |
| 20 | 60.06 | 4.47 | 2319 | 13800 | 23 | 305 | 956 | 2314 | 11489 |
| 21 | 6.90 | 3.88 | 228 | 16062 | 79 | 864 | 1134 | 5064 | 10995 |
| 22 | 19.74 | 6.71 | 1141 | 20862 | 108 | 1876 | 1840 | 9992 | 10871 |
| 23 | 61.83 | 7.79 | 3007 | 16132 | 52 | 813 | 1114 | 4678 | 11455 |
| 24 | 20.25 | 8.38 | 1103 | 21420 | 122 | 2193 | 1436 | 10940 | 10479 |
| 25 | 37.11 | 9.52 | 1525 | 15522 | 39 | 531 | 798 | 3156 | 12365 |
| 26 | 108.29 | 11.56 | 5381 | 8729 | 26 | 361 | 1127 | 2727 | 5998 |
| 27 | 20.21 | 4.29 | 521 | 9363 | 16 | 523 | 858 | 3046 | 6313 |
| **Total** | - | - | 68834 | 420641 | 1874 | 61459 | 81079 | 338159 | 70659 |

**
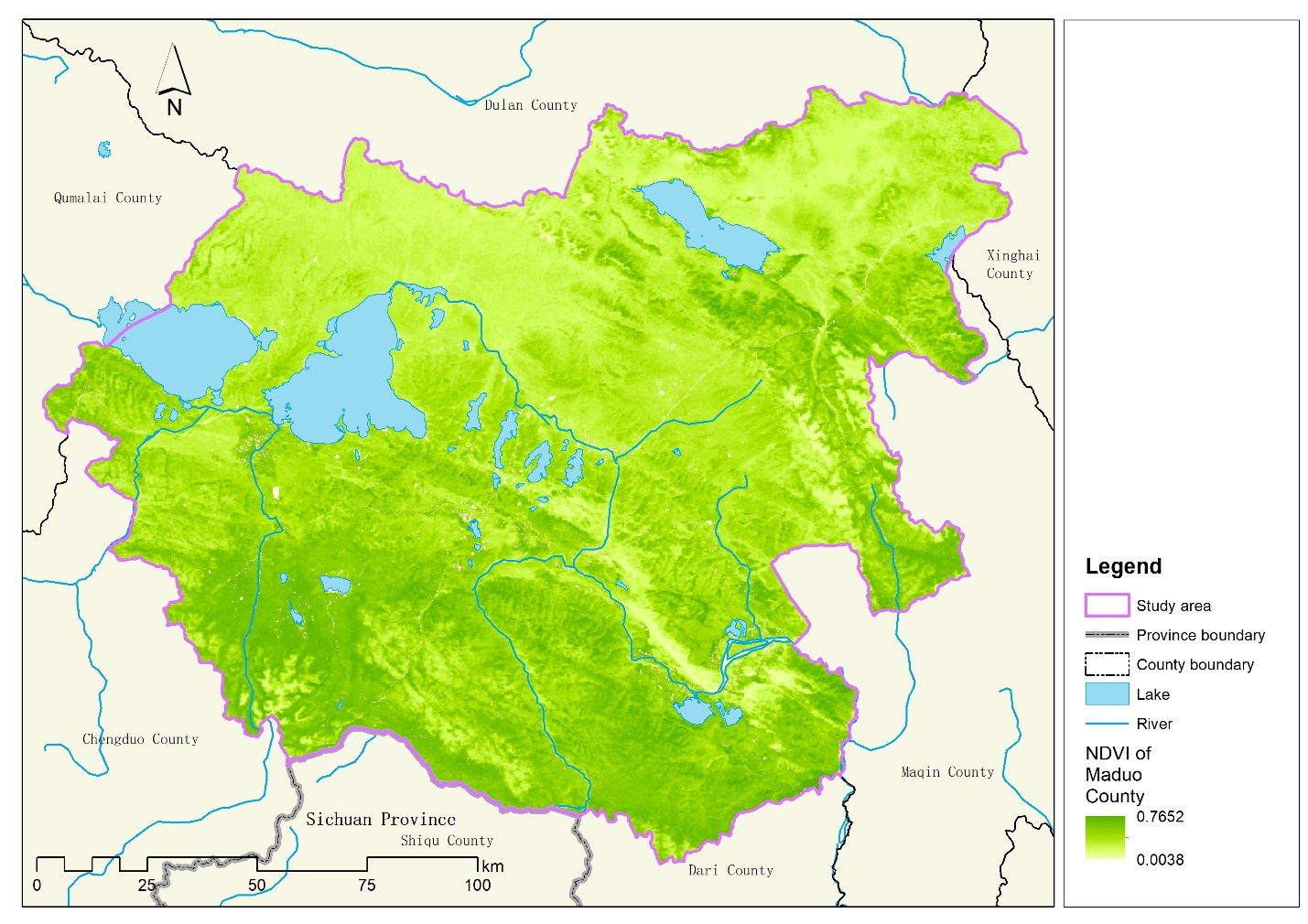
**

**Supplementary Figure S1** Spatial distribution of normalized difference vegetation index (NDVI) values in Maduo County in 2015. The map was generated using ArcGIS 10.2, https://desktop.arcgis.com/en/.
